# Modeling future cliff-front waves during sea level rise and implications for coastal cliff retreat rates

**DOI:** 10.1038/s41598-024-57923-0

**Published:** 2024-04-02

**Authors:** H. Matsumoto, M. E. Dickson, W. J. Stephenson, C. F. Thompson, A. P. Young

**Affiliations:** 1 Scripps Institution of Oceanography, University of California San Diego, San Diego, USA; 2 The University of Auckland, Auckland, New Zealand; 3 University of Otago, Dunedin, New Zealand

**Keywords:** Natural hazards, Physical oceanography

## Abstract

It is often assumed that future coastal cliff retreat rates will accelerate as global sea level rises, but few studies have investigated how SLR (sea level rise) might change cliff-front wave dynamics. Using a new simple numerical model, this study simulates the number and type (breaking, broken, or unbroken) of cliff-front waves under future SLR scenarios. Previous research shows breaking waves deliver more energy to cliffs than broken waves, and unbroken waves generate minimal impact. Here, we investigated six cliff-platform profiles from three regions (USA, New Zealand, and UK) with varied tidal ranges and wave climates. Model inputs included 2013–2100 hindcast/forecast incident wave height and tidal water level, and three future SLR scenarios. Results show the number of both cliff-front breaking and broken waves generally increase for a high-elevation (relative to tide) cliff-platform junction. In contrast, breaking/broken wave occurrence decrease by 38–92% for a near-horizontal shore platform with a low-elevation cliff-platform junction under a high SRL scenario, leading to high (96–97%) unbroken wave occurrence. Overall, results suggest the response of cliff-front waves to future SLR is complex and depends on shore platform geometries and SLR scenarios, indicating that future cliff retreat rates may not homogeneously accelerate under SLR.

## Introduction

It is generally expected that coastal cliff erosion rates will accelerate as global sea level rises, with support for this assertion found in numerous modeling studies^1–6^. For instance, at two relatively slowly retreating (historic retreat rates of ~ 4–6 cm/year) sites in the UK, Shadrick et al.^7^ used cosmogenic radionuclides (^10^Be) and topographic profile data combined with numerical modeling to detect a relationship between the Holocene rate of SLR (sea level rise) and the rate of coastal cliff retreat. They suggest that accelerating SLR will lead to an increase in rates of cliff retreat by up to an order of magnitude by 2100. Numerical modeling of rapidly retreating (historic retreat rates of tens of cm/year) glacial till cliffs in the UK suggests that retreat rates will likely accelerate with SLR, although there may be a significant lagged response^3^. An ensemble model for southern California cliffs suggests that future cliff retreat rates could more than double compared to mean historical rates^6^.

Intuitively, cliff erosion rates should increase with SLR, because deeper water in front of a cliff will result in decreased nearshore wave dissipation. Implicit within this statement, however, is an assumption that cliff erosion rates are primarily controlled by wave erosion. Recent studies show a quantitative link between wave power and cliff(-base) erosion rates^8,9^. The nature of this association is clarified through three years of weekly laser-scan observations from Del Mar, California, that reveal cliff-toe erosion is correlated with wave impact height and duration, whereas erosion higher on the profile is better correlated with rainfall^10^. At this site, Clow et al.^11^ measured and modeled cosmogenic ^10^Be concentrations across the shore platform fronting the eroding cliff, and found that cliff retreat rates over the last 2000 years were similar to modern erosion rates, consistent with relatively stable SLR rates over the same period. These results suggest that in southern California, waves provide a mechanism by which SLR can contribute to cliff erosion rates.

Other studies suggest a contrasting or less equivocal relationship between SLR and cliff erosion rates. For example, Swirad et al.^12^ measured cosmogenic ^10^Be concentrations across a sandstone shore platform in North Yorkshire, UK, revealing approximately constant cliff retreat rates (~ 4.5 cm/year) through time and no direct relationship to sea level changes over centennial to millennial timescales. Using a simple exploratory model, Ashton et al.^13^ conceptually proposed various cliff response behaviors ranging from responsive to unresponsive to changes in the rate of SLR. Furthermore, an extensive literature documents the extent to which a myriad of weathering processes contribute to, and in some cases, dominate the rate of cliff erosion^14,15^. For instance, cliff failure frequently occurs without waves impacting directly on the cliff^16,17^. Recently, Dietze et al.^18^ showed that during the monitoring of a cliff on the Jasmund Peninsula of Rügen, Germany, marine processes did not trigger cliff failure, and wetting of the chalk was the most significant process.

While deeper water does result in decreased nearshore wave dissipation in front of coastal cliffs, the actual energy delivered into cliffs is complex, resulting from intricate wave dissipation and refraction processes on the beaches and shore platforms^19,20^, and highly complex wave breaking dynamics and impact regimes at the cliff face^21^. Field measurements over a single tidal cycle show that subtle water level changes can rapidly transition cliff-toe wave energy spectra from long- to short-wave dominated^22^, and wave breaking regimes on the cliff face from broken, to breaking and unbroken wave impacts, resulting in variability in cliff ground shaking (a potential proxy for erosional damage) that varies by an order of magnitude^21^.

Complex depth-controlled process interactions such as these prompted Dickson et al.^23^ to caution that cliff erosion rates may not uniformly accelerate with SLR. The prospect of slower cliff erosion under SLR seems intuitively unlikely, but if future SLR is very fast, it is plausible that the rate of rise in water level might vastly exceed the erosional morphological response of the cliff, and inundation of the lower part of the cliff might transform both the weathering and hydrodynamic regime on the shore platform and cliff toe. For example, deeper water conditions at the cliff base could change wave cliff interaction from waves breaking on to the cliff to more reflective conditions. These types of breaking wave regime changes have not yet been investigated in detail.

Here, we explore the nature of SLR-driven modulation of cliff-front wave conditions using a new simple numerical model. In a laboratory experiment, Sunamura^24^ found cliff erosion and shore platform development differed in response to breaking, broken, and (standing) unbroken wave actions, and we followed this concept. Our model tracks the number of breaking, broken, and unbroken waves likely to occur in front of a cliff, given three SLR-scenarios and six shore platform geometries found in three locations with different tidal regimes (Auckland—New Zealand, San Diego—USA, and Vale of Glamorgan—Wales, UK).

## Methods

### Model concept

To explore the influence of future SLR on wave impacting coastal cliffs, we use a simple cross-shore model that simulates cliff-front wave type (breaking, broken, and unbroken) depending on incident waves and water level variability owing to tides and projected sea levels between 2013 and 2100. This study uses ‘wave type’ to specifically indicate breaking, broken and unbroken wave type (as opposed to plunging, spilling, and surging ‘wave type’). Existing models^25–27^ do not solve wave shoaling, but estimate cliff-front wave type and cliff impacting wave force by identifying wave breaking locations relative to the cliff-toe location and calculating wave type/force as a function of the distance between the wave breaking and cliff-toe locations. The model in this study follows a similar approach but is forced by higher temporal resolution hindcast/projected oceanographic data to simulate detailed future cliff-front wave dynamics. Following Sunamura^24,28^, we used the number and type of cliff-front waves as a proxy for cliff erosion potential.

**Figure 1:**
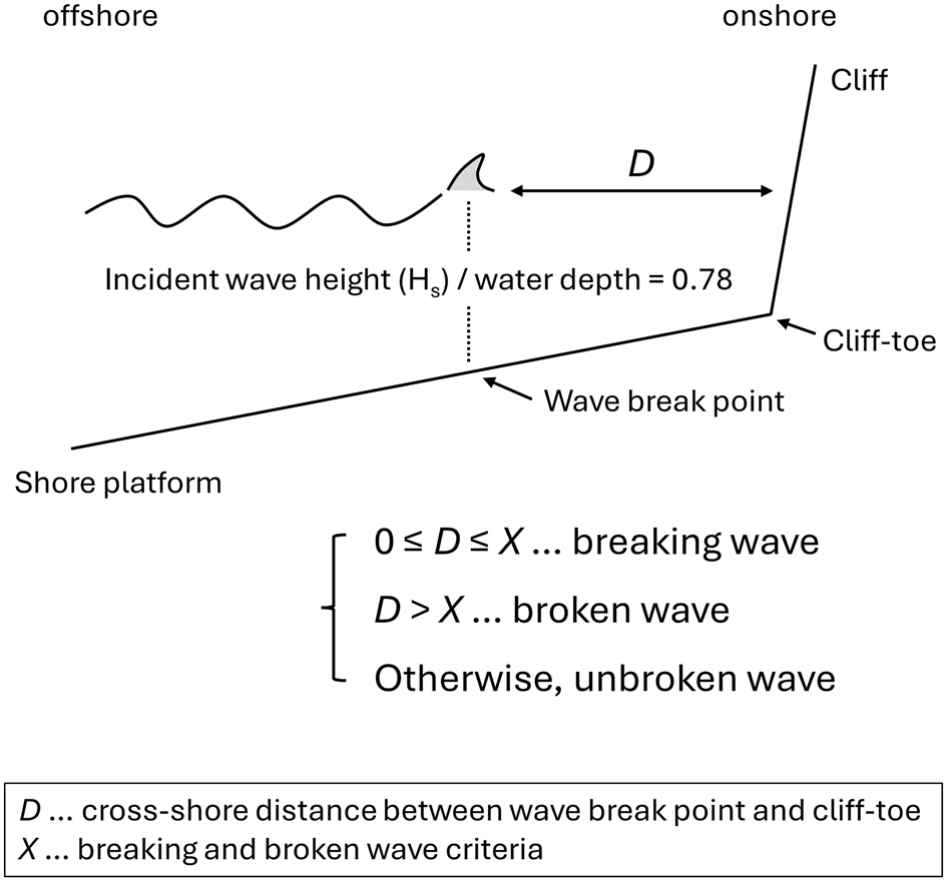
Schematic diagram of the wave type classification in relation to the cross-shore distance from the wave break point to the cliff-toe (*D*), and breaking and broken wave criteria (*X*).

For each time step the wave break location and its (cross-shore) distance from the cliff-toe (denoted as *D* in this study) were estimated assuming a wave break condition of incident wave height/water depth = 0.78^29^ (Fig. 1). The water depth for each time step considered tidal water level and the SLR scenario. The cross-shore distance from the wave break point to the cliff-toe (*D*) was then used to determine cliff-front wave type (breaking or broken) using breaking/broken wave criteria (denoted as *X*), where *D* is smaller or larger than *X*, the wave type is breaking or broken, respectively. When the water level is too deep to trigger wave breaking (break point ratio > 0.78), unbroken waves reflect off the cliff. While existing models used a single threshold value (of *X*) for the breaking and broken wave classification, this study tested a range of threshold values (see result section).

Numerous simplifications are involved in any simple model study^30^. In this work we neglect effects associated with wave refraction/diffraction, wave setup/runup, and wave reforming. Profile changes owing to erosion such as mechanical wave erosion and weathering processes are not modeled, and mobile sediment that could accelerate or reduce erosion^31^ are not considered. We discuss the potential importance of these simplifying assumption in our discussion.

### Model inputs and parameters

**Figure 2:**
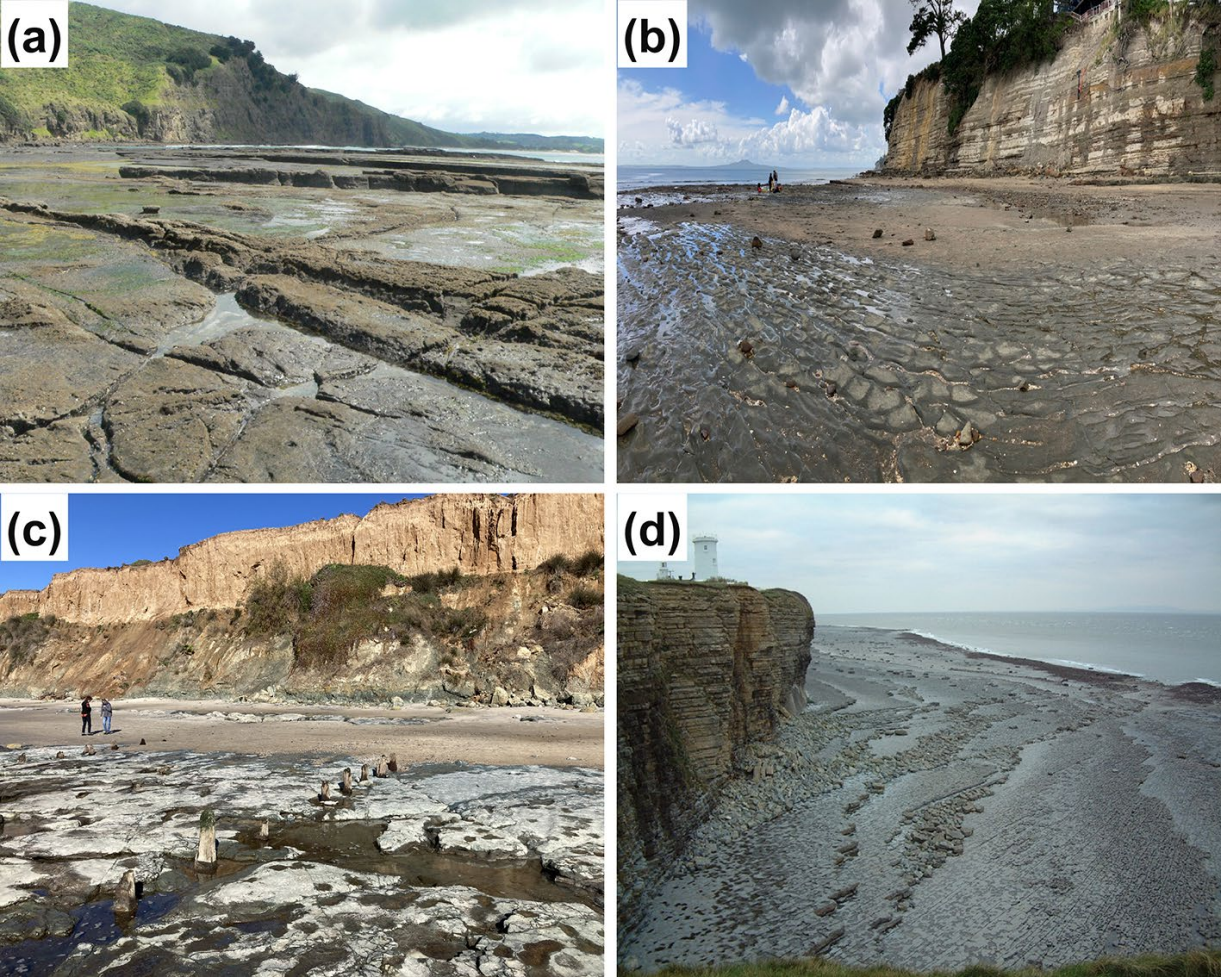
Example photos of (**a**) AK1, (**b**) AK3, (**c**) SD2, and (**d**) VG.

**Figure 3:**
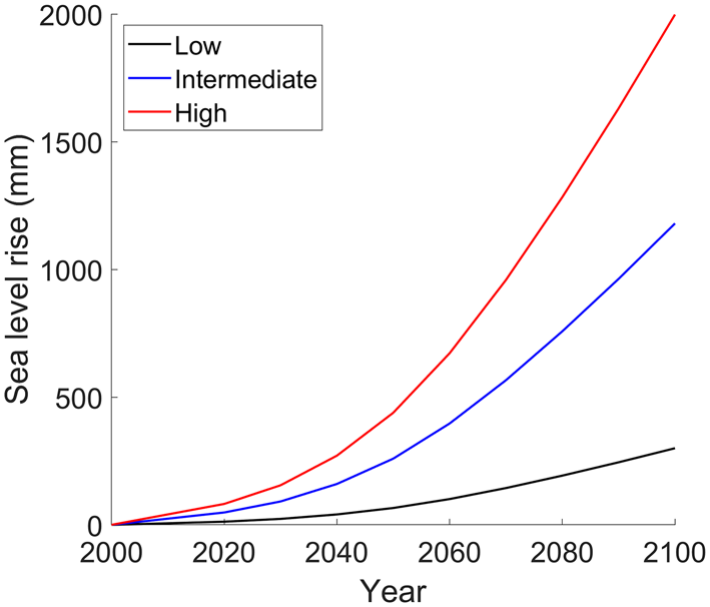
Low, intermediate, and high (median quartile) SLR scenarios from Sweet et al.^36^.

Model inputs include six simplified cliff profiles (three from Auckland, two from San Diego, and one from Vale of Glamorgan, Table 1 and Fig. 2), three SLR scenarios, and hourly time series of hindcast/forecast tidal water level and incident wave height (*H*_s_). The SLR scenarios include low, intermediate, and high (median quantile) from Sweet et al.^36^ that estimate year 2100 sea levels of 300, 1180, and 1998 mm (relative to year 2000), respectively (Fig. 3). This study also tested regional SLR scenarios and found general similarities qualitatively between the results using Fig. 3 and regional SLR scenarios. For each SLR scenario, models were run at hourly time steps from 2013 to 2100.

**Table 1:** Summary of study site cliff-forming lithology, cliff erosion rate, and wave climatology.

Location	Cliff-forming lithology	Cliff erosion rate [m/year]	Wave climatology
Okakari point, Auckland (AK1)	Flysch (volcanic-rich) with alternating mudstone and sandstones*	< 0.02^32^	Intermediate wave energy with occasional tropical storm events
Red beach, Auckland (AK2)	Flysch (volcanic-poor) with alternating siltstones and sandstones*	< 0.02^32^	Low wave energy with occasional tropical storm events
Rothesay bay, Auckland (AK3)	Flysch (volcanic-poor) with alternating siltstones and sandstones*	< 0.02^32^	Low wave energy with occasional tropical storm events
Sunset cliff, San Diego (SD1)	Cretaceous sedimentary overlaid by Pleistocene terrace deposits^33^	0.06–0.43^34^	Seasonal winter-high and summer-low wave energy
Del Mar, San Diego (SD2)	Eocene sedimentary overlaid by weakly cemented sandy Pleistocene terrace deposits^33^	0.03–0.10^34^	Seasonal winter-high and summer-low wave energy
Nash point, Vale of Glamorgan (VG)	Jurassic Blue Lias Limestone, alternating bands of organic-rich, finely laminated shales and limestones^35^	0.06–0.08^35^	High energy storm wave

*General rock hardness: AK1 > AK2 > AK3

For the Auckland (AK) sites, modeled incident wave height (*H*_s_) with three-hour resolution was obtained based on the work of Albuquerque et al.^37^ at a location in ~ 41 m water depth and ~ 30–50 km from the AK sites for 1986–2005, 2026–2045, and 2081–2100 time periods (https://uoa-eresearch.github.io/waves/projections.html). Because temporal gaps (i.e. 2006–2025, 2046–2080) exist, existing hindcast/forecast data were used to fill the gaps (i.e., 1986–2005, 2026–2045, and 2081–2096 wave data was copied for 2006–2025, 2046–2065, and 2066–2080 time periods, respectively). Modeled hourly tidal levels were obtained using 2000–2020 tidal data from a location about 17–54 km from the AK sites (https://tides.niwa.co.nz/) applied to matlab UTide function^38^.

Modeled incident wave height (*H*_s_, 8–11 m water depth) with three-hour resolution from Hegermiller et al.^39^ was used for the San Diego (SD) sites. Hourly forecast tidal levels were obtained for the La Jolla tide gauge (http://tidesandcurrents.noaa.gov), located 5–16 km from the SD sites. Three-hour time series of *H*_s_ were linearly interpolated to match the hourly tidal data.

Modeled hourly incident wave height (*H*_s_) was obtained from a location (~ 15 m water depth) 19.6 km from the Vale of Glamorgan (VG) site between 2006 and 2100^40^. Modeled hourly tidal levels were obtained using 1990–2011 tidal data from a Hinkley tidal station (https://www.bodc.ac.uk/data/hosted_data_systems/sea_level/uk_tide_gauge_network/, about 34 km from the VG site) applied to matlab UTide function.

### Profile shapes

**Figure 4:**
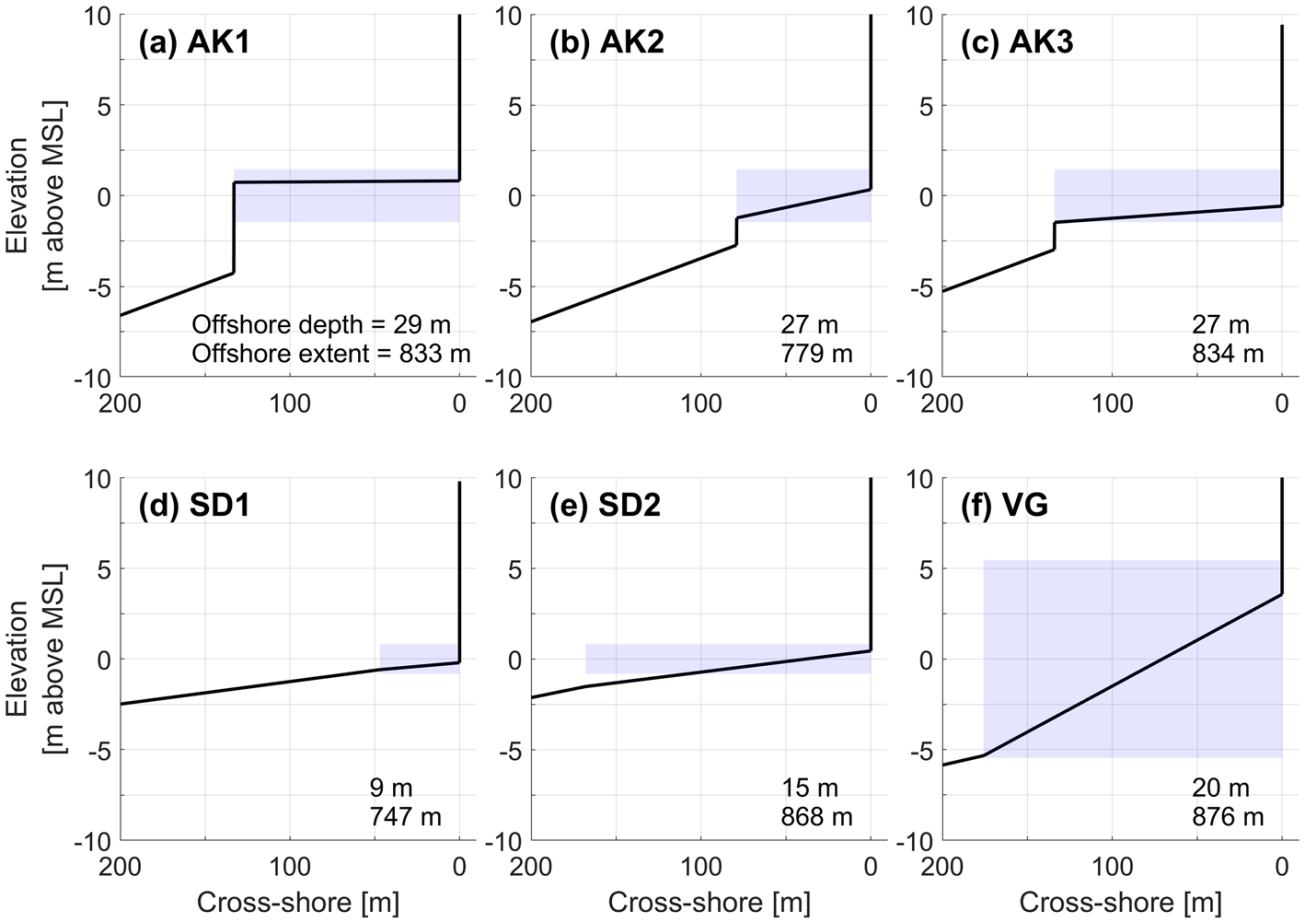
Model cross-shore profiles from (**a**–**c**) Auckland (AK), New Zealand, (**d**,**e**) San Diego (SD), USA, and (**f**) Vale of Glamorgan (VG), Wales, UK. Table 2 shows profile details. Model vertical (= 90 deg) cliffs are located at 0 m cross-shore position. Blue shades show spring tidal range (vertical) and shore platform cross-shore extent (horizontal). MSL denotes mean sea level. The numbers in the bottom right corners are offshore depth below MSL (top) and offshore extent from cliff-toe position (bottom).

The simplified cross shore profiles represent typical cliff-platform settings including: cliff-fronted sloping (> 1 deg) and near-horizontal (< 1 deg) shore platforms in 1.6–2.9 m tidal settings; and sloping (> 1 deg) shore platforms in a 10.9 m tidal setting (Fig. 4 and Table 2). These shore platform geometries broadly represent variability in platform characteristics that occur globally^41,42^, but local variability exists between sites in factors such as nearshore bathymetry, platform roughness, and sediment cover. Offshore profiles were not available for the AK and VG profiles, and 2- and 4-degree offshore slopes were selected, respectively. Note that the deeper offshore profile slope did not influence the modeled wave breaking characteristics in general, and these slopes provide illustrative context only (Fig. 4). The profile cross-shore resolution was 10 cm.

**Table 2:** Metrics for the model profiles shown in Fig. 4.

Location	Shore platform width (m)	Shore platform slope (deg)	Shore platform mean elevation (m, relative to MSL)	Cliff-platform junction elevation (m, relative to MSL)	Tidal range (m)	Offshore slope (deg)
AK1	133	0.03	0.78	0.81	2.9	2
AK2	79	1.13	− 0.44	0.34	2.9	2
AK3	134	0.38	− 1.02	− 0.57	2.9	2
SD1	47	0.47	− 0.04	− 0.21	1.6	0.7
SD2	168	0.67	− 0.50	0.44	1.6	1.2
VG	176	2.90	− 0.55	3.56	10.9	4

## Results

### Model sensitivity to breaking and broken wave classification criteria

This study determined wave type using the cross-shore distance from the wave break point to the cliff base (*D*), and breaking and broken wave classification criteria (*X*, Fig. 1). To examine the model sensitivity to *X*, this study tested a range of *X* values (Table 3).

**Table 3:** Test cases for breaking and broken wave classification criteria. For cases 1–4, *X* depended on incident wave height (*H*_s_), which was held constant in each time step because the model does not solve wave shoaling.

	*X* [m]	Breaking wave	Broken wave
Case1	0.5*H*_s_	0 ≤ *D* ≤ 0.5*H*_s_	*D* > 0.5*H*_s_
Case2	*H*_s_	0 ≤ *D* ≤ *H*_s_	*D* > *H*_s_
Case3	2*H*_s_	0 ≤ *D* ≤ 2*H*_s_	*D* > 2*H*_s_
Case4	5*H*_s_	0 ≤ *D* ≤ 5*H*_s_	*D* > 5*H*_s_
Case5	1	0 ≤ *D* ≤ 1	*D* > 1
Case6	2	0 ≤ *D* ≤ 2	*D* > 2
Case7	5	0 ≤ *D* ≤ 5	*D* > 5
Case8	10	0 ≤ *D* ≤ 10	*D* > 10

**Figure 5:**
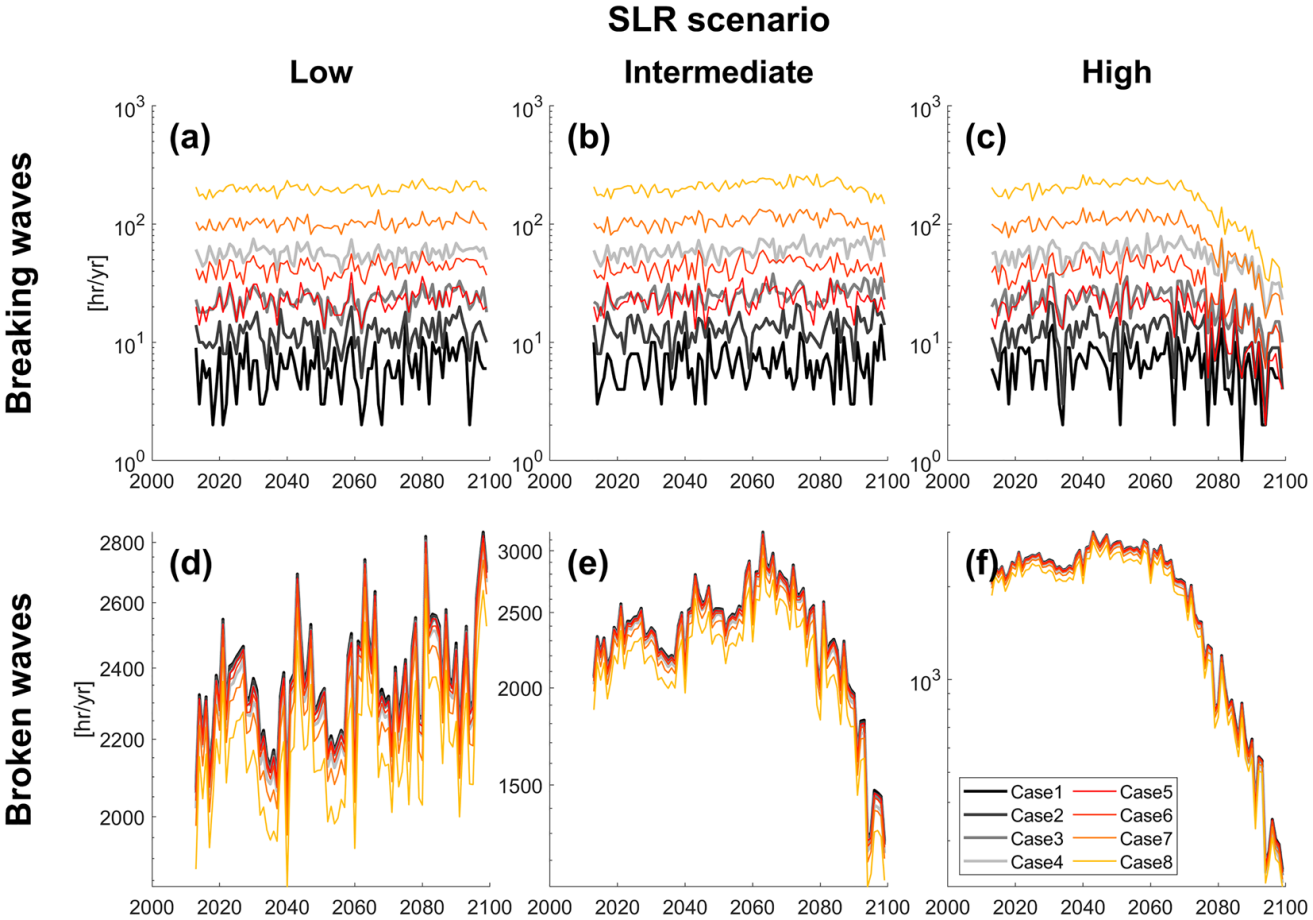
Hours of (**a**–**c**) breaking and (**d**–**f**) broken waves in front of the cliff versus time for AK3 profile for low (left panels), intermediate (center), high (right) SLR scenarios. Color lines show results with different *X* values shown in Table 3.

For AK3 profile, the different *X* values influenced cliff base wave conditions, with more breaking waves and less broken waves for larger *X* (e.g., Case1 vs. Case4, Fig. 5). However, temporal changes in the number of cliff-front breaking/broken wave hours per year were similar for different test cases. For example, cliff-front breaking wave hours per year remained almost constant through time for all the test cases with the low SLR scenario (Fig. 5a). Similarly, both cliff-front breaking and broken wave hours peaked in ~ 2060–2070 and then steadily decreased with time for all test cases with the high SLR scenarios (Fig. 5c,f). Note, similar model behavior was observed for other profiles (supplementary data). Overall, the results demonstrate temporal changes in cliff-front waves are relatively insensitive to the breaking and broken wave classification criteria.

### Cliff-front waves in response to SLR

**Figure 6:**
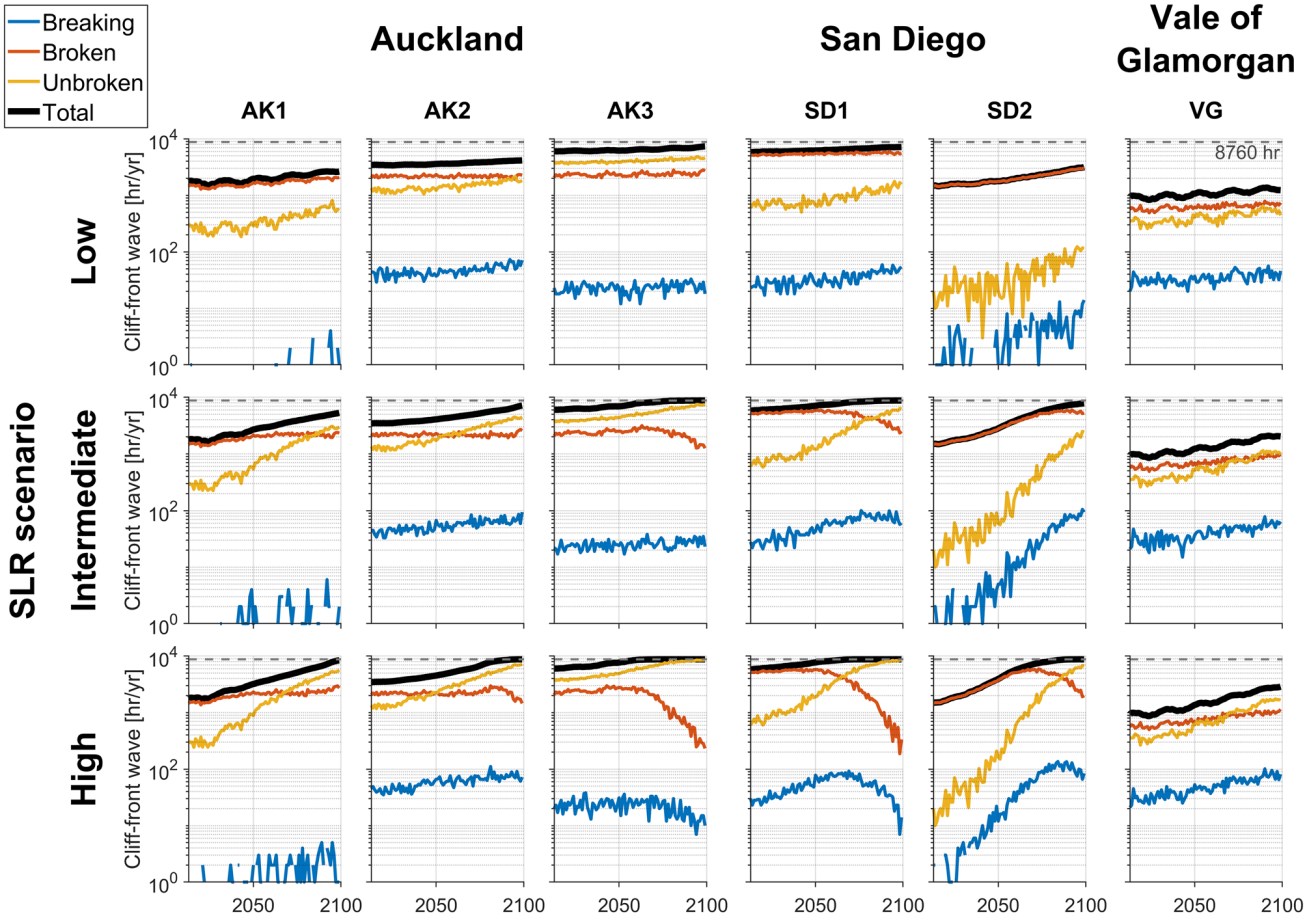
Time series of cliff-front breaking (blue), broken (orange), unbroken (yellow), and total (black) wave hours per year for Auckland (AK1/AK2/AK3), San Diego (SD1/SD2), and Vale of Glamorgan (VG) profiles for low (top panels), intermediate (middle), and high (bottom) SLR scenarios. Dashed gray shows the maximum wave hours per year (8760 = 365 days × 24 h). Case3 wave type classification criteria used (Table 3). The vertical axes use a log scale.

In total, 18 simulations (six profiles and three SLR scenarios) were conducted to examine changes in cliff-front waves in response to SLR. For all profiles and SLR scenarios, more broken (orange, Fig. 6) and unbroken (yellow, Fig. 6) waves occurred in front of the cliff than breaking waves (blue, Fig. 6). For all profiles and the high SLR scenario, unbroken waves occurred most frequently between 2085 and 2100. The proportion of breaking waves relative to broken/unbroken waves was the largest for VG profile. The total number of cliff-front waves (= breaking + broken + unbroken, black line, Fig. 6) were higher for AK3 and SD1 profiles (~ 6000–8760 h per year), and lower for VG profile (~ 1000–3000 h per year) because high-elevation (relative to tide) cliff-platform junctions (Table 2) prevented waves from reaching the cliff.

A range of temporal changes in the number and type of cliff-front waves occurred, with generally larger changes for higher SLR scenarios (Fig. 6 and Table 4). Both cliff-front total and unbroken waves increased through time for all profiles and SLR scenarios, particularly for AK1 and SD2 profiles with a near-horizontal shore platform and a relatively high-elevation (relative to tide) cliff-platform junction (Table 2), where the total number of cliff-front waves increased 47–110% and 350–480% for the low and high SLR scenarios, respectively. Similarly, for SD2 profile, both cliff-front breaking, and unbroken waves considerably increased particularly for the intermediate and high SLR scenarios (~ 70–80 times increase for breaking wave, and 140–410 times increase for unbroken wave, Table 4). In contrast, for AK3 and SD1 profiles with a near-horizontal shore platform and a relatively low-elevation cliff-platform junction (Table 2), the increase in cliff-front total waves was relatively small (20–49%), whereas cliff-front breaking and broken waves exhibited more complex temporal changes, particularly with the high SLR scenario (Fig. 6). For instance, after an initial increase until ~ 2060–2070, the number of both cliff-front breaking and broken waves declined sharply, resulting in an overall decrease of 38–42% and 87–92%, respectively. For AK2 and VG profiles with a sloping shore platform, the temporal changes in cliff-front breaking and broken waves were relatively small ranging 19–151% and -22–85%, respectively.

**Table 4:** Percentage change in cliff-front breaking, broken, unbroken, and total wave hours per year from 2013 to 2100. Mean values of 2013–2018 and 2095–2100 were used for the calculations. Breaking waves for AK1 profile are excluded owing to the infrequent occurrence (≤ 6 h per year, Fig. 5). Int denotes the intermediate SLR scenario.

SLR scenario	Breaking wave			Broken wave			Unbroken wave			Total		
	Low	Int	High	Low	Int	High	Low	Int	High	Low	Int	High
AK1	–	–	–	35	50	84	110	892	1693	47	187	350
AK2	45	85	67	4	16	− 22	45	237	440	19	99	152
AK3	22	26	− 42	19	− 37	− 87	21	93	121	20	45	45
SD1	85	153	− 38	8	− 48	− 92	126	754	1061	22	49	49
SD2	1150	7176	8340	104	263	51	657	14,290	40,594	110	415	480
VG	19	83	151	24	63	85	38	182	348	29	109	189

## Discussion

**Figure 7:**
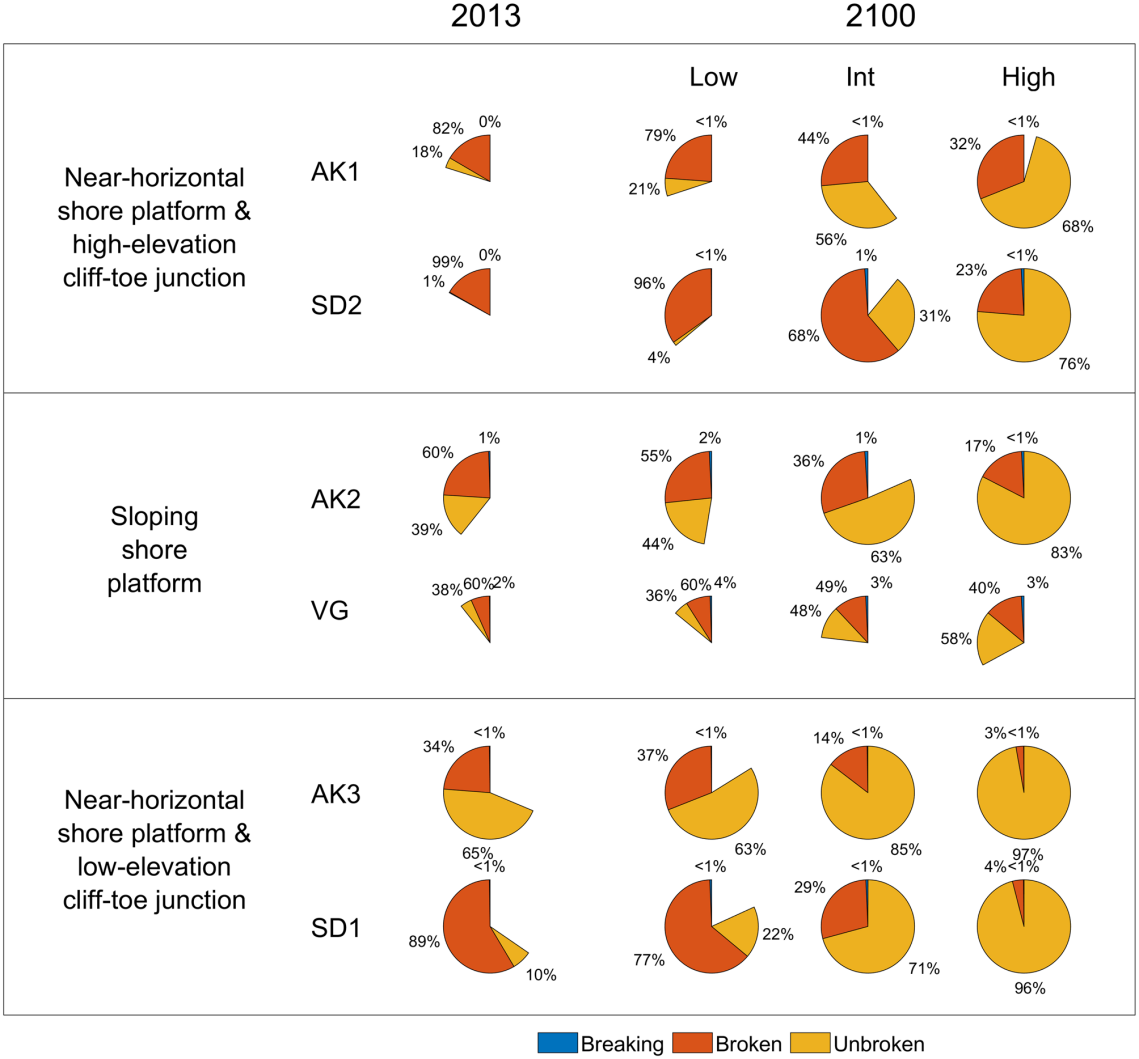
The number of cliff-front waves (indicated by partial pie size) and the proportion of cliff-front breaking, broken and unbroken wave hours (indicated by numbers) in 2013 and 2100.

Results show that the number and type of cliff-front waves vary depending on shore platform geometries and SLR scenarios (Table 4 and Fig. 7). For a near-horizontal shore platform with a high-elevation (relative to tide) cliff-platform junction (such as AK1 which has been interpreted as a shore platform that likely formed under mid-Holocene higher sea level^43^, and SD2) where platform elevations are generally high, most waves break in front or on the shore platform with a relatively few cliff-front breaking and unbroken waves (82–99% relative broken wave occurrence, Fig. 7). Increasing sea levels considerably increase wave impact (47–480% increase in total cliff-front waves, Table 4) including cliff-front breaking and broken wave actions owing to the increase in cliff-front water level, likely increasing future cliff erosion rates. Similar general increase in cliff-front breaking and broken waves with time also occur for a sloping shore platform (e.g., AK2 and VG, with a generally high-elevation cliff-platform junction) but more moderately (Table 4), because the steeper slope prevents rapid landward shift of breaker zones in response to SLR compared to a near-horizontal shore platform. In contrast, for a near-horizontal platform with a low-elevation (relative to tide) cliff-platform junction (e.g., AK3 and SD1), wave impact is relatively active owing to relatively deep water (see the size of partial pies in Fig. 7). In addition, at these sites with increasing sea levels, total cliff-front waves remain relatively unchanged (20–49% increase between 2013 and 2100, Table 4), while cliff-front breaking and broken waves decrease considerably (38–42% and 87–92% respectively, for the high SLR scenario, Table 4), shifting the cliff-front wave regime to higher (71–97% for the intermediate and high SLR scenarios, Fig. 7) unbroken wave occurrence and possibly reducing future cliff erosion rates.

Cliff Stability Indexes (CSI)^44–46^ rely on the calculation of wave energy delivery for cliff erosion estimates, and typically assume a linear relationship between energy delivery from wave impacts and increased SLR and wave storminess, resulting in increased future cliff erosion risk. Other studies used the number of hours that (total) water level exceeds cliff toe elevation^47–49^ as cliff erosion factors. Our results show that, even with gradually increasing sea levels (Fig. 3), the temporal changes in cliff-front breaking and broken waves in response to SLR can be complex (e.g., an initial increase followed by steady decrease for AK3 and SD1 profiles with the high SRL scenario, Fig. 6). Such complex changes in cliff-front wave environments and associated cliff erosion potential could be usefully incorporated in future cliff erosion assessments.

Our results should be interpreted with caution, as our approach does not account for morphological change over the timescale of the model simulations (~ 100 years). This assumption may be reasonable for cliffs with relatively slow historical retreat rates, but rapidly eroding soft rock cliffs are expected to retreat tens of meters over the next century, and morphological changes will influence wave energy dissipation on the shore platform and cliff and hence cliff erosion rates. For instance, Dickson et al.^2^ showed using a numerical model that future recession rates of soft rock coasts in the northeast UK can decrease locally owing to sediments distributed from neighboring rock coast erosion that buffered cliff-toe from erosion. Hence, a model that incorporates morphological feedbacks is preferred to project erosion rates for these cliff types^50^.

The present model does not account for beaches fronting cliffs, water level changes driven by processes (e.g., wave setup) other than tide and sea levels, stochastic nature of future wave conditions, and weathering and biological processes^51,52^. Cliffs fronted by beaches are common^53^, and Earlie et al.^54^ demonstrated how wave runup on steeper beaches increases cliff exposure to wave energy. Thompson et al.^21^ found subtle changes in water level can dramatically alter wave breaking conditions in front of a cliff, while changes in the position of still water level may also alter the effective zone of abrasive sediments at the cliff base^55^. Vitousek et al.^56^ demonstrated the importance of ensemble wave forcing considerations (instead of a deterministic single time series) in sandy shoreline modeling, although there have been few cliff erosion studies considering ensemble wave forcing. The suite of weathering processes operating on coastal cliffs should change under accelerating SLR, but it is not yet clear whether weathering and biological processes will accelerate or reduce erosion rates in response to SLR. More advanced modeling could incorporate these factors to better understand if and how future SLR might alter the contributions of weathering and wave processes at sites where weathering currently dominates.

Cliff retreat rates are generally expected to increase as future SLR accelerates. This modeling study illustrates how varied shore platform geometry (particularly cliff-platform junction elevation and platform slope) and SLR scenarios can drive variable temporal changes in the number of cliff-front breaking, broken and unbroken waves. Breaking waves can deliver more energy to the cliff than broken waves, while unbroken waves generate relatively minimal wave impacts^24,57,58^. Our results reinforce recent speculation^23^ that changes in cliff-front wave types in response to future SLR are complex, and future cliff erosion are unlikely to homogeneously accelerate with SLR owing to important changes in wave-energy expenditure against cliffs associated with shifting cliff-front wave regimes. Where cliff erosion is driven by waves (rather than subaerial processes), retreat rates may increase or decrease depending on changes in wave environments at the cliff base.

### Supplementary Information

Supplementary Figures.

## Data Availability

The datasets generated during and/or analyzed during the current study are available from the corresponding author on reasonable request.
