# Supplementary material for: Modeling future cliff-front waves during sea level rise and implications for coastal cliff retreat rates
Source: Sci Rep. 2024 Apr 2;14:7810. doi: 10.1038/s41598-024-57923-0 (PMC10987572; doi:10.1038/s41598-024-57923-0)
Supplement: Supplementary file 1 — Supplementary Figures. [file 41598_2024_57923_MOESM1_ESM.docx]

**Modeling future cliff-front waves during sea level rise and implications for coastal cliff retreat rates**

**Matsumoto, H.^1,*^, Dickson, M.E.^2^, Stephenson, W.J.^3^, Thompson, C.F.^1^, Young, A.P.^1^**

^1^Scripps Institution of Oceanography, University of California San Diego, USA

^2^The University of Auckland, New Zealand

^3^University of Otago, New Zealand

^*^himatsumoto@ucsd.edu


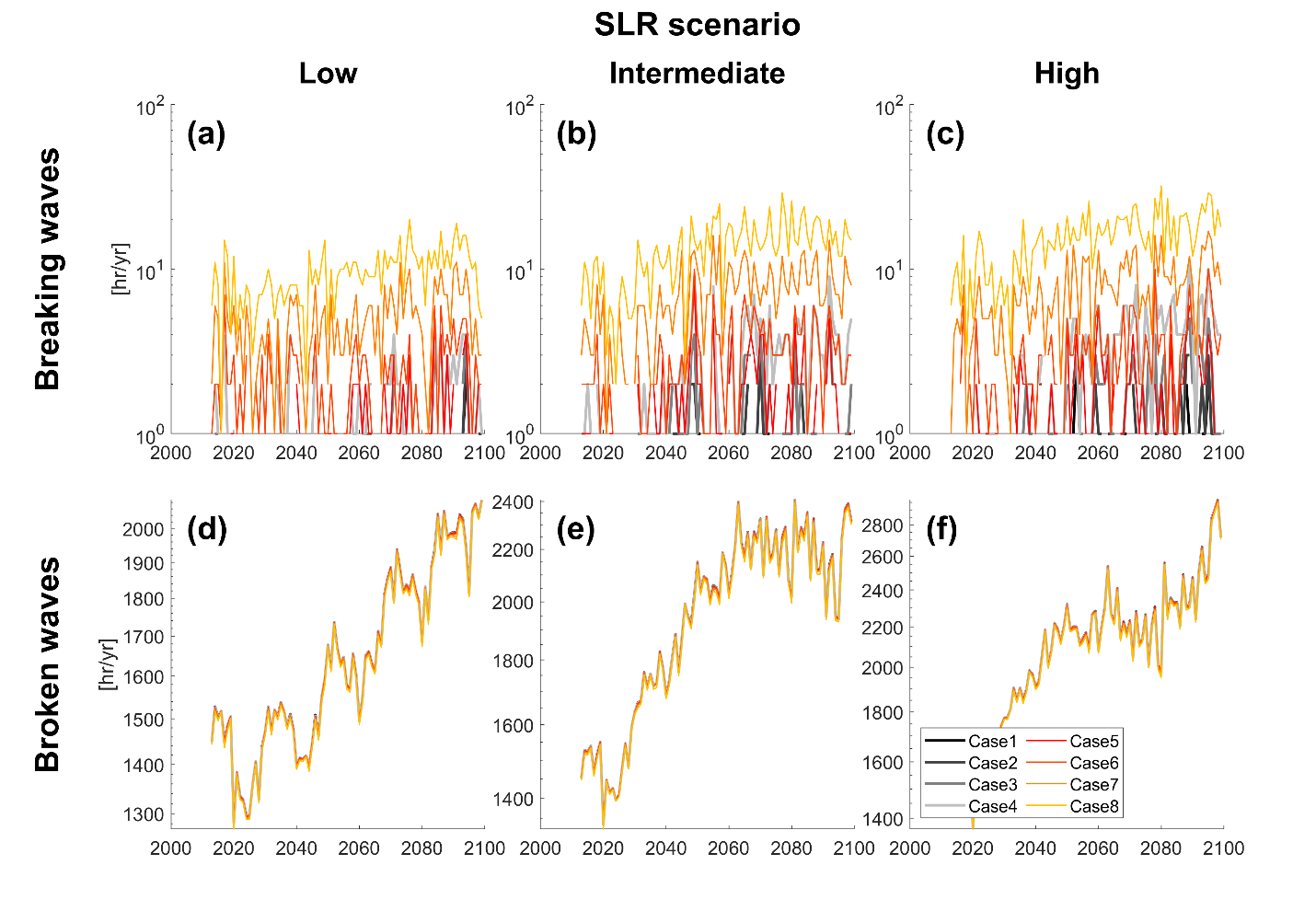


Figure S1. Hours of (a-c) breaking and (d-f) broken waves in front of the cliff versus time for AK1 profile for low (left panels), intermediate (center), high (right) SLR scenarios. Color lines show results with difference *X* values shown in Table 3.


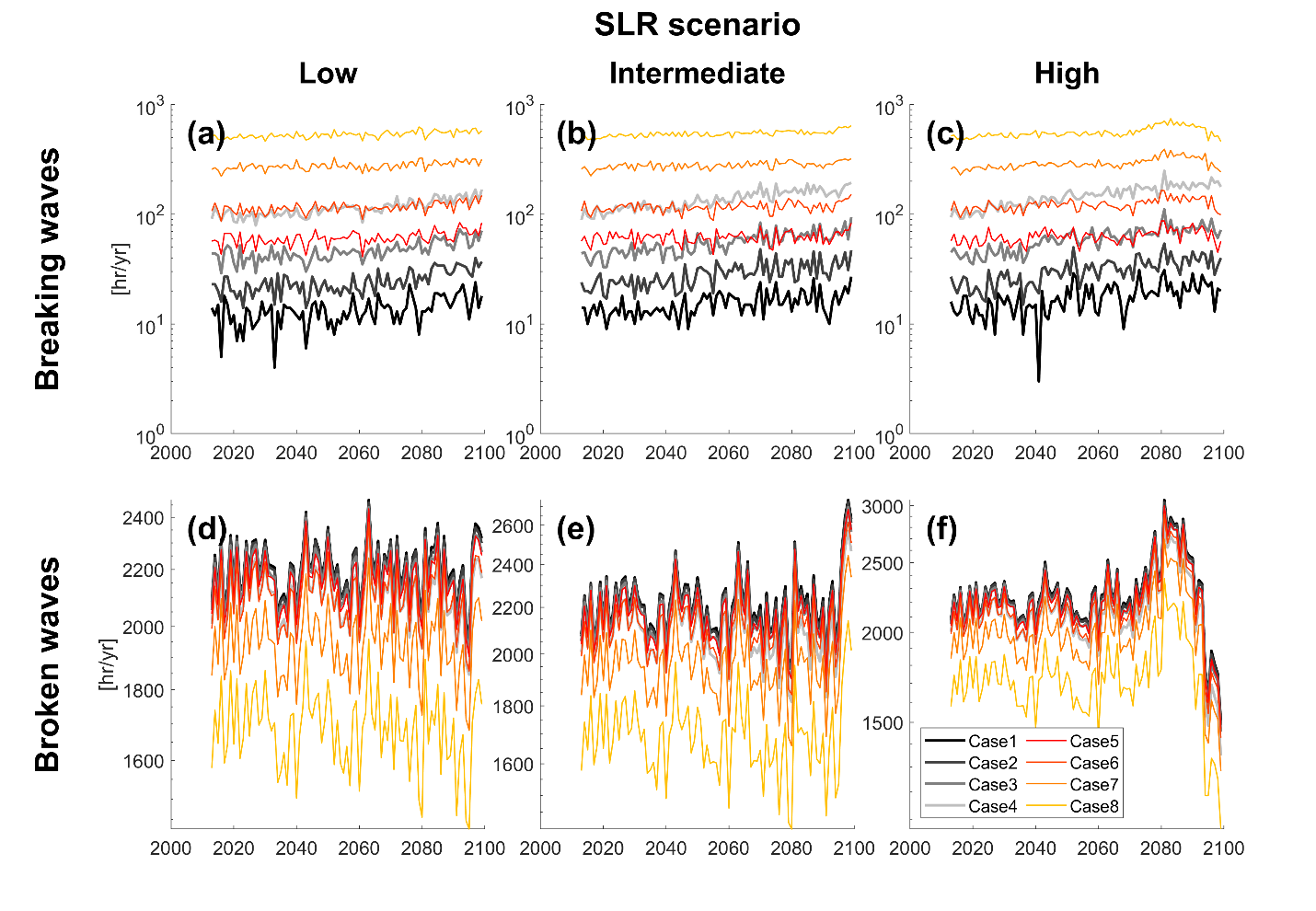


Figure S2. Hours of (a-c) breaking and (d-f) broken waves in front of the cliff versus time for AK2 profile for low (left panels), intermediate (center), high (right) SLR scenarios. Color lines show results with difference *X* values shown in Table 3.


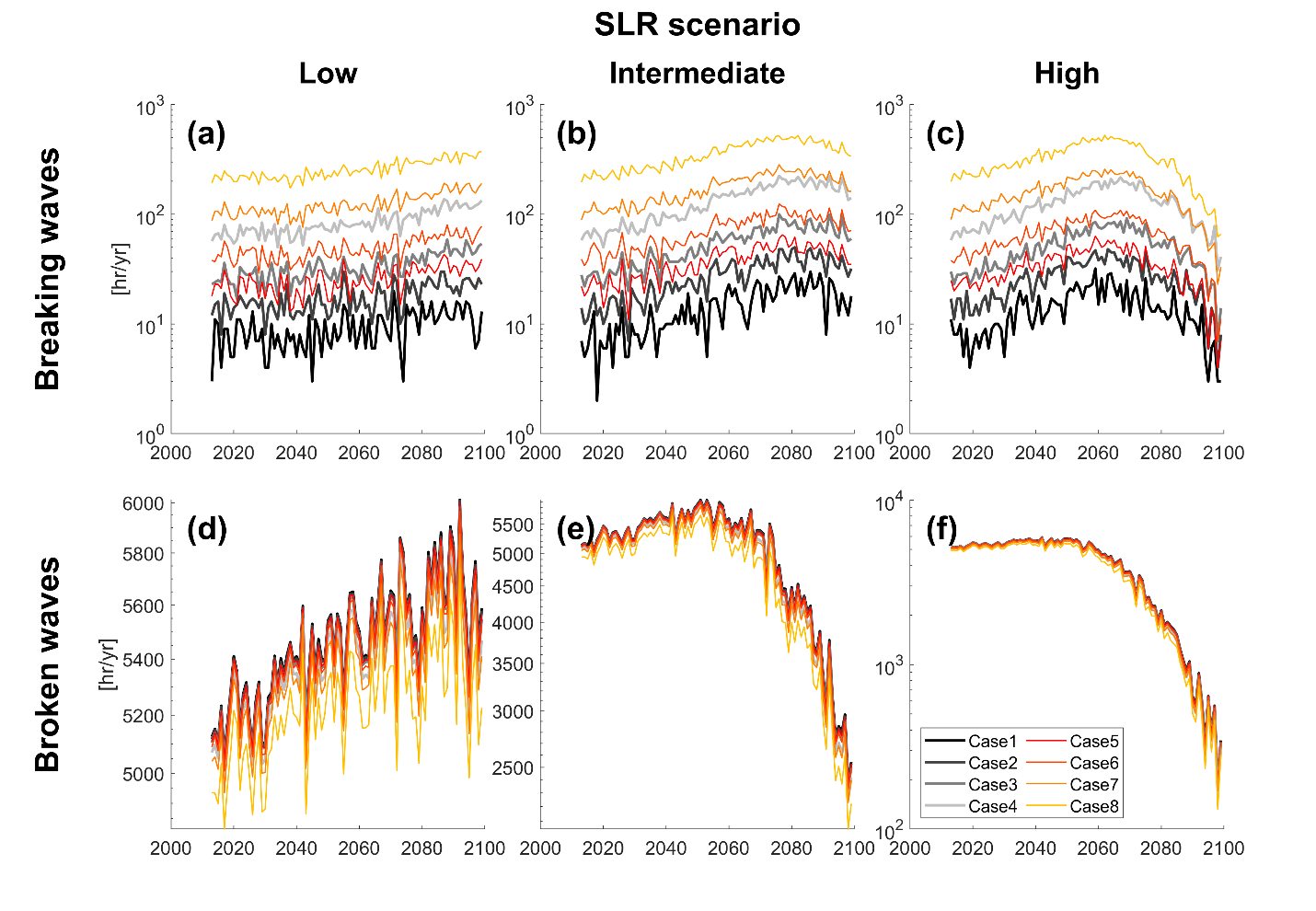


Figure S3. Hours of (a-c) breaking and (d-f) broken waves in front of the cliff versus time for SD1 profile for low (left panels), intermediate (center), high (right) SLR scenarios. Color lines show results with difference *X* values shown in Table 3.


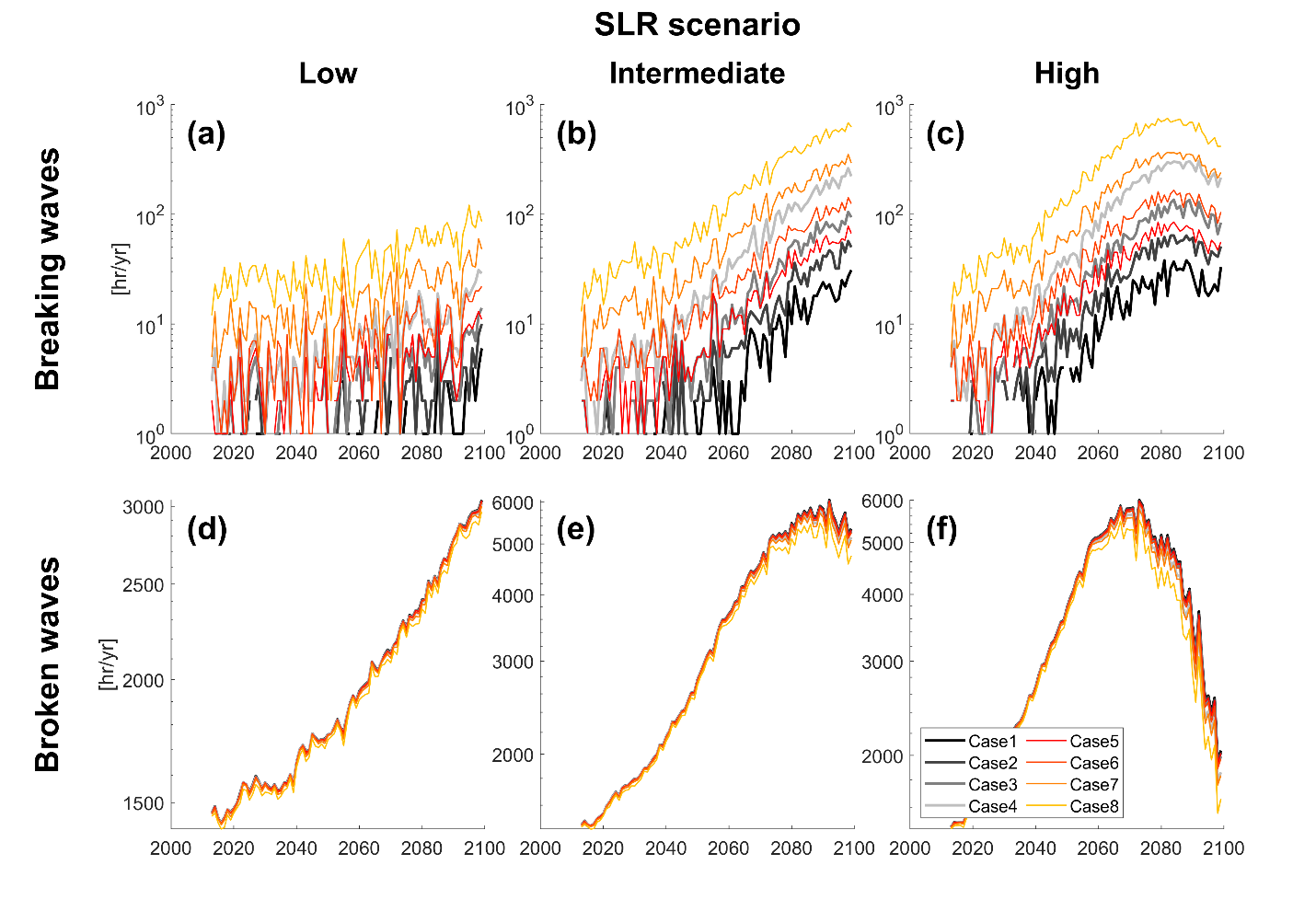


Figure S4. Hours of (a-c) breaking and (d-f) broken waves in front of the cliff versus time for SD2 profile for low (left panels), intermediate (center), high (right) SLR scenarios. Color lines show results with difference *X* values shown in Table 3.


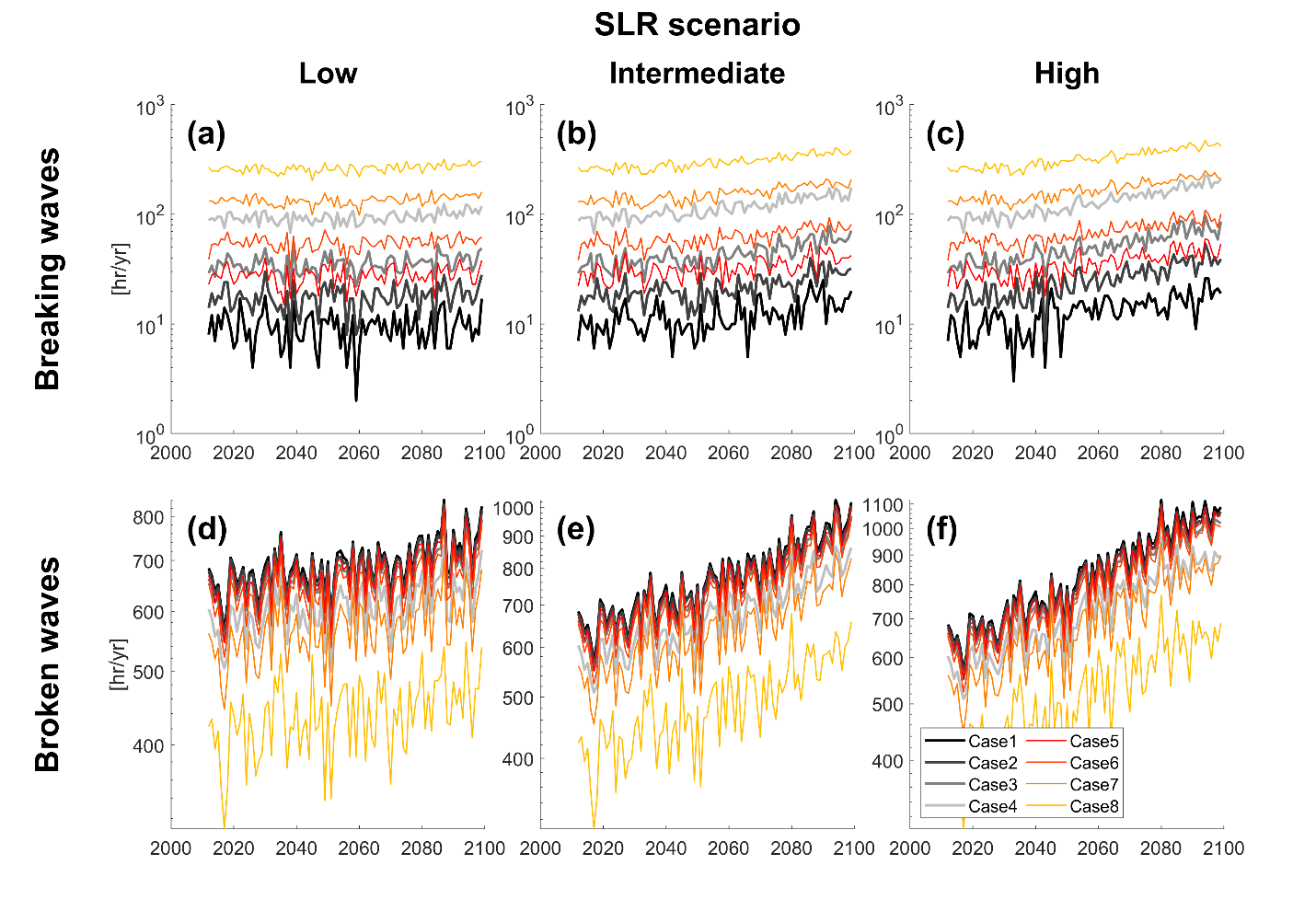


Figure S5. Hours of (a-c) breaking and (d-f) broken waves in front of the cliff versus time for VG profile for low (left panels), intermediate (center), high (right) SLR scenarios. Color lines show results with difference *X* values shown in Table 3.
